# Prevalence and associated factors of internet gaming disorder among secondary school students in rural community, Thailand: a cross-sectional study

**DOI:** 10.1186/s13104-019-4862-3

**Published:** 2020-01-06

**Authors:** Pawan Taechoyotin, Puttipun Tongrod, Tanawat Thaweerungruangkul, Nitchapat Towattananon, Pitch Teekapakvisit, Chanapon Aksornpusitpong, Wichapa Sathapornpunya, Nutchar Hempatawee, Ram Rangsin, Mathirut Mungthin, Phunlerd Piyaraj

**Affiliations:** 1 Fourth Year Medical Cadet, Phramongkutklao College of Medicine, Bangkok, Thailand; 2 Department of Military and Community Medicine, Phramongkutklao College of Medicine, Bangkok, Thailand; 3 Department of Parasitology, Phramongkutklao College of Medicine, 317/5 Ratchavithi Rd, Ratchathewi, Payathai, Bangkok, 10400 Thailand

**Keywords:** Internet gaming disorder, Prevalence, Risk factors, Adolescent, Rural community, Thailand

## Abstract

**Objective:**

Internet gaming disorder (IGD) is an emerging mental problem for adolescent that has been increasingly piqued interest over the last decade. Although many studies have been conducted, very few studies have studied populations with low technological access. Therefore, this study aimed to estimate the prevalence and associated factors of internet gaming disorder among secondary school students in an area with a comparatively lower technological access in a large community sample. We used Chachoengsao province, Thailand as our sample area with a comparatively lower access to technology. This cross-sectional study was conducted during December 2017–January 2018 among 12 secondary schools in Chachoengsao province, Thailand.

**Results:**

5.4% of the 5497 subjects were positive for IGD. The associated factors found were male gender (odds ratio (OR) = 1.4), not living with both parents (OR = 1.75), use of online dating (OR = 1.53), being bullied at school (OR = 1.51), depression (OR = 1.92), anxiety (OR = 1.62) and stress (OR = 3.57) after being adjusted for age, family composition, failure of an exam, limit of internet use, use of online dating, bully perpetration, bully victimization, cyber bully perpetration, cyber bully victimization, suicidal ideation, use of alcohol, depression, anxiety, and stress.

## Introduction

Over the last decade, the number of internet users has significantly risen. Thailand in particular, is no exception. There has also been an increase in reports of people being preoccupied with the internet to the extent of having a negative impact on their lives. Internet addiction is the term used to identify this kind of behavior [1]. According to Young, internet addiction can be categorized in five specific subtypes including cyber-sexual addiction, cyber-relationship addiction, net compulsions, information overload and computer addiction (obsessive computer game playing) [2]. Despite having five subtypes of internet addiction, obsessive computer game playing has received the most attention over the years.

The term internet gaming addiction was first reported in 2004 [3]. In the following years, studies were conducted to further explore the nature of internet gaming addiction. In 2012, a systematic review included neuroimaging studies providing an explanation on the pathology and mechanisms among those with internet addiction and also internet gaming addiction [4]. In 2013, the official term, “internet gaming disorder” (IGD), was established by the American Psychological Association (APA) with the definition of “repetitive use of Internet-based games, often with other players, that leads to significant issues with functioning”. The term was also added to the Diagnostic and Statistical Manual of Mental Disorders, Fifth Edition (DSM-5). However, it was labeled as a condition warranting further research. The diagnostic criteria for IGD consists of nine items, having at least 5 within 1 year warrants the diagnosis. The IGD-20 test was later developed as a valid and reliable tool for diagnosing IGD [5].

Factors associated with IGD include functional and dysfunctional impulsivity, belief self-control, anxiety, pursuit of desired appetitive goals, money spent on gaming, weekday game time, offline community meeting attendance, gaming community membership [6], game genre [7], age of initiation [8], poorly functioning family [9], high levels of psychological distress, use of alcohol, suicidal ideation [10], having distinct problematic thoughts about gaming [11], higher neuroticism, decreased conscientiousness and low extraversion [12]. Although the direction of these associations have yet to be concluded, studies are still ongoing to clarify these issues.

Studies have been conducted in various countries on the prevalence of IGD. The prevalence in a systematic review of 50 studies ranged from 0.7 to 27.5% [13]; the author also noted that comparison might be difficult due to the diverse tools used to identify IGD. Few studies included in this systematic review used the IGD-20 tool. Moreover none did specify whether the area of study was urban or rural.

Up to the present, many studies on IGD have been conducted. However, there have been few studies who have aimed to estimate the prevalence of IGD in a rural area of a developing country where there is a technological gap compared to urban areas. Thailand is an area where such a gap exists [14]. Therefore, this study aimed to estimate the prevalence of IGD and also identify its associated factors in rural area of a developing country.

## Main text

### Study design

A cross-sectional study of prevalence of IGD was conducted from December 2017 to January 2018 among secondary schools in Chachoengsao Province, Thailand. Chachoengsao is a sample of a rural area in Thailand. Our target population was the age of 12–18 years. So we used students currently studying in secondary schools (grade 7–12) in Chachoengsao as our population. There were a total of 43 secondary schools in Chachoengsao, 12 were private schools while the rest were public schools. A stratified sampling based on type of school and size was used to select the schools. Our sampling yielded 10 public schools and 2 private schools. Students currently studying in the selected schools were recruited via advertisement from the school director. Informed consent was required for each participant to partake in the study. Eligible participants comprised Thai nationality, students attending the 12 secondary school in Chachoengsao Province, Thailand. Information of general characteristics, IGD and mood disorder screening (depression, anxiety and stress) were collected by self-administered standardized questionnaires.

### Measures

Both online and paper questionnaires contained 45 independent variables, divided in 6 parts. The first part comprised general characteristics, the second part described father and mother status while the third part covered internet and game assessment. The fourth part involved the internet gaming disorder test (IGD-20 Test) translated to Thai and the fifth part measured depression, anxiety and stress using the short form of the Depression Anxiety Stress Scales (DASS-21) Thai version. The sixth part comprised questions about bullying and sexual intercourse.

The main outcome of this study focused on internet gaming disorder. Other variables in addition to the main outcome involved depression, anxiety and stress. General characteristics, also in the questionnaire were age, sex, family income, debt status, weight, height, school location, school type, bullying factors and internet access.

#### Internet gaming disorder test (IGD-20 Test)

The main outcome was measured using the IGD-20 Test translated to Thai consisting of 20 questions. Each question has scores from 1 to 5, minimum and maximum scores are 20 and 100 and the cut point is 71. Questions covered salience, mood modification, withdrawal symptoms, conflict and relapse.

### Statistical analysis

Descriptive statistics including percentage, mean, median and mode were used to evaluate distribution before further statistic evaluation. We conducted univariate regression to compare the IGD group to the control group in terms of general characteristics, psychological symptom, internet assessment and bullying. The potential independent risk factors with p-value < 0.2 were included in multivariate logistic regression analysis, which was performed to determine the risk factors associated with IGD. The analysis was performed using SPSS 22.0. A p-value < 0.05 was considered statistically significant in the analysis.

### Results

Of those 9649 students from the 12 schools, 6000 (62.1%) were enrolled in the study. After excluding missing data, only 5497 (91.6%) were applicable. Of 5497 participants the prevalence of IGD-20 positive was 5.4%. Psychological symptoms were around 49.2% of those reporting depressive symptoms. In all, 55.4% accounted for anxiety symptoms and 28% for stress symptoms. The sex of participants was 44.9% male and 55.1% female. Totally, 51.8% of the participants had grade point average (GPA) lower than 2.50 (average grade lower than C+), and 68.4% had failed an exam in the past 12 months. The proportion of schools inside and outside the city district was 55.2 and 44.8%, respectively. Type of school was divided in public (67.4%) and private (32.6%). Altogether, 98.8% of participants had internet access and 80.7% have played online games. In this population 36% of gamers have topped up in the game ranging from 9 to 100,000 Thai baht (THB) (0.3–3000 US dollar (USD)). Fully, 87.3% of participants used their phone as the most used device, 22.7% had been cyber-bullied and 17.8% had bullied others (Table 1).

**Table 1:** General characteristic of population in the study of prevalence and associated factor of Internet gaming disorder (IGD) among secondary school students in rural community, Thailand

General characteristic	Internet gaming disorder (IGD)		Total n (%)	p-value
	No n (%)	Yes n (%)		
Age (years): median (Q1–Q3)	15.0 (14.0–17.0)	14.0 (13.0–16.0)	15.0 (14.0–17.0)	< 0.001
Sex				< 0.001
Female	1935 (43.2)	134 (56.5)	2069 (43.9)	
Male	2541 (56.8)	103 (43.5)	2644 (56.1)	
Failed an exam at least once in life				0.067
No	1645 (36.2)	72 (30.4)	1717 (35.9)	
Yes	2895 (63.8)	165 (69.6)	3060 (64.1)	
Failed an exam in past 12 months				0.508
No	1453 (32.0)	71 (30.0)	1524 (31.9)	
Yes	3086 (68.0)	166 (70.0)	3252 (68.1)	
Repeater in past 12 months				0.023
No	4462 (98.3)	230 (96.2)	4692 (98.2)	
Yes	79 (1.7)	9 (3.8)	88 (1.8)	
Smoking				< 0.001
No	4054 (89.3)	196 (81.7)	4250 (88.9)	
Yes	487 (10.7)	44 (18.3)	531 (11.1)	
Alcohol drinking				0.001
No	2615 (57.6)	127 (52.9)	2742 (57.4)	
Social drinking	1854 (40.8)	102 (42.5)	1956 (40.9)	
Regular drinking	70 (1.5)	11 (4.6)	81 (1.7)	
Use of addictive substances				0.490
No	4411 (97.0)	231 (96.3)	4642 (97.0)	
Yes	135 (3.0)	9 (3.8)	144 (3.0)	
Average sleeping time (hours/day)				< 0.001
≤ 4 h	260 (5.8)	34 (14.3)	294 (6.2)	
5–8 h	3313 (73.3)	161 (67.9)	3474 (73.0)	
≥ 9 h	947 (21.0)	42 (17.7)	989 (20.8)	
Type of school				0.731
Public	2860 (62.7)	153 (63.7)	3013 (62.7)	
Private	1705 (37.3)	87 (36.3)	1792 (37.3)	
Family income (THB)				0.007
< 15,000	2060 (45.4)	130 (54.4)	2190 (45.8)	
> 15,000	2478 (54.6)	109 (45.6)	2587 (54.2)	
Sufficiency of family income				0.460
Yes	4017 (88.2)	208 (86.7)	4225 (88.2)	
No	535 (11.8)	32 (13.3)	567 (11.8)	
Living with				< 0.001
Father and mother	3380 (74.2)	145 (60.7)	3525 (73.6)	
Other	1173 (25.8)	94 (39.3)	1267 (26.4)	
Internet access				0.484
No	53 (1.2)	4 (1.7)	57 (1.2)	
Yes	4500 (98.8)	236 (98.3)	4736 (98.8)	
Average of internet use (hours/day)				< 0.001
Median (Q1–Q3)	7.0 (4.0–11.0)	10.0 (5.0–14.0)	7.0 (4.0–12.0)	
Internet gaming use				< 0.001
No	874 (19.2)	22 (9.2)	896 (18.7)	
Yes	3672 (80.8)	217 (90.8)	3889 (81.3)	
Average of internet gaming use (hours/day)				< 0.001
Median (Q1–Q3)	3.0 (1.0–5.0)	5.0 (3.0–8.0)	3.0 (2.0–5.0)	
Paid for internet gaming				< 0.001
No	2833 (65.4)	95 (41.9)	2928 (64.2)	
Yes	1500 (34.6)	132 (58.1)	1632 (35.8)	
Amount paid for internet gaming (THB)				0.006
Median (Q1–Q3)	500.0 (150.0–1600.0)	700.0 (300.0–3000.0)	500.0 (170.8–2000.0)	
The device most used for internet access				0.046
Computer	524 (11.5)	38 (15.8)	562 (11.7)	
Smartphone	3958 (86.8)	201 (83.8)	4159 (86.7)	
Tablet	68 (1.5)	0 (0.0)	68 (1.4)	
Other	9 (0.2)	1 (0.4)	10 (0.2)	
Restraint of internet usage				0.399
No	2756 (60.5)	138 (57.7)	2894 (60.3)	
Yes	1801 (39.5)	101 (42.3)	1902 (39.7)	

After univariate and multivariate logistic regression analysis was conducted, factors associated with internet gaming disorder exhibiting a p-value < 0.05 included being male; odds ratio (OR) = 1.4 (95% confidence interval (95% CI) 1.06–1.85), not living with both father and mother; OR = 1.75 (95% CI 1.32–2.31), use of online dating; OR = 1.53 (95% CI 1.01–2.32), being bullied at school; OR = 1.51 (95% CI 1.05–2.18), depression; OR = 1.92 (95% CI 1.35–2.74), anxiety; OR = 1.62 (95% CI 1.11–2.35) and stress; OR = 3.57 (95% CI 2.59–4.97) (Table 2).

**Table 2:** Logistic regression analysis of risk factors associated with internet gaming disorder (IGD) among secondary school students in rural community, Thailand

Associated factors	Internet gaming disorder		Univariate analysis			Multivariate analysis		
	No (n%)	Yes (n%)	Crude OR	95% CI	p-value	Adjusted OR	95% CI	p-value
Age (years): median (Q1–Q3)	15.0 (14.0–17.0)	14.0 (13.0–16.0)	0.90	0.84–0.97	0.004	0.95	0.88–1.02	0.163
Sex								
Female	2541 (96.1)	103 (3.9)	1.00			1.00		
Male	1935 (93.5)	134 (6.5)	1.71	1.31–2.22	< 0.001	1.43	1.07–1.91	0.015
Living with								
Father and mother	4157 (95.3)	204 (4.7)	1.00			1.00		
Other	396 (91.9)	35 (8.1)	1.87	1.43–2.44	< 0.001	1.67	1.25–2.24	0.001
Failed an exam								
No	1453 (95.3)	71 (4.7)	1.00			1.00		
Yes	3086 (94.9)	166 (5.1)	1.30	0.98–1.73	0.068	1.23	0.90–1.67	0.194
Restraint of internet usage								
No	2756 (95.2)	138 (4.6)	1.00			1.00		
Yes	1801 (94.7)	101 (5.3)	1.12	0.86–1.46	0.399	1.12	0.84–1.49	0.446
Use of online dating								
No	4283 (95.4)	208 (4.6)	1.00			1.00		
Yes	270 (89.4)	32 (10.6)	2.44	1.65–3.61	< 0.001	1.42	9.91–2.21	0.125
School bullying victim								
No	3107 (96.6)	111 (3.4)	1.00			1.00		
Yes	1440 (91.8)	129 (8.2)	2.51	1.93–3.26	< 0.001	1.57	1.08–2.30	0.020
School bully perpetrator								
No	3151 (96.4)	119 (3.6)	1.00			1.00		
Yes	1395 (92.0)	121 (8.0)	2.30	1.77–2.98	< 0.001	1.19	0.81–1.75	0.367
Cyberbullying victim								
No	3557 (95.7)	158 (4.3)	1.00			1.00		
Yes	971 (92.2)	82 (7.8)	1.90	1.44–2.51	< 0.001	0.95	0.65–1.39	0.795
Cyberbully perpetrator								
No	3745 (95.7)	168 (4.3)	1.00			1.00		
Yes	767 (91.4)	72 (8.6)	2.09	1.57–2.79	< 0.001	1.18	0.79–1.76	0.417
Suicidal ideation								
No	4411 (95.0)	231 (5.0)	1.00			1.00		
Yes	135 (93.8)	9 (6.3)	1.97	1.30–2.97	0.001	0.82	0.51–1.30	0.391
Depression								
No	3240 (97.5)	83 (2.5)	1.00			1.00		
Yes	1299 (89.3)	156 (10.7)	4.69	3.57–6.17	< 0.001	1.93	1.34–2.77	< 0.001
Anxiety								
No	2659 (97.8)	59 (2.2)	1.00			1.00		
Yes	1880 (91.3)	180 (8.7)	4.32	3.20–5.82	< 0.001	1.65	1.13–2.41	0.010
Stress								
No	4010 (97.8)	128 (3.1)	1.00			1.00		
Yes	529 (82.7)	111 (17.3)	6.57	5.02–8.61	< 0.001	3.42	2.46–4.76	< 0.001
Type of school attending								
Public	2860 (94.9)	153 (5.1)	1.00			1.00		
Private	1705 (95.1)	113 (5.5)	0.95	0.73–1.25	0.731	0.96	0.74–1.34	0.735
Consumes alcoholic beverages								
No	2615 (95.4)	127 (4.6)	1.00			1.00		
Yes	1924 (94.5)	113 (5.5)	1.21	0.93–1.57	0.152	0.88	0.65–1.18	0.390

### Discussion

The prevalence among secondary school students in a rural community of Thailand was 5.4%, which could be considered slightly higher than average in other studies. However, the difference was quite minimal suggesting that sociocultural differences may not be associated with internet gaming disorder [15]. Being male was significantly associated with internet gaming disorder. Women may show better executive control than men when facing gaming cues, which may provide resiliency against developing IGD [16]. Another study identified the differences of the brain in men and women with IGD [17]. Not living with both parents was also significantly associated with internet gaming disorder. Having a non-intact family is associated with poorer family functioning, lower maternal and paternal behavioral control [18]. This increases the risk of developing IGD [19, 20]. The use of online dating reflects higher social anxiety [21, 22], thus increasing the likelihood of internet addiction [23]. Being bullied at school may indicate an individual’s low self-esteem [8, 19, 24, 25]. This also serves as a criteria for internet gaming disorder. As for depression stress and anxiety, the causal relationship is unclear due to limitations in this study design. The associations may be interpreted both ways. The individual may feel depressed, stressed or anxious by the events in the real world and may choose to use the virtual world (where they feel safe and secure) to escape these feelings. Another interpretation may be that internet gaming disorder causes depression, stress and anxiety due to its impact on an individual’s neuro-circuitry [26–28]. Further studies employing a different design that would be able to explain the causal relationship should be conducted to make a more valid conclusion.

The strengths of this study were the relatively large sample size, inclusion of comprehensive risk factors, our standardize definitions of diagnoses, the recently updated information and to our knowledge this constitutes the first study conducted in Thailand.

### Conclusion

In conclusion, this research demonstrated the most recent estimated prevalence and the associated factors of Internet gaming disorder among adolescents in a rural community of Thailand. A qualitative study should be conducted in the future to better understand the relationships to mental health outcomes at the national level. Although the prevalence of Internet gaming disorder among Thai secondary school students may not be high when compared with other published studies, the global trend shows a continuous increased prevalence. Therefore, preventive measures should be taken. Interventions should particularly target those with increased risk of developing this disorder.

## Limitation of the study

Because the study employed a cross-sectional design, causal relationships remains unclear. In addition, the data obtained may have been subjected to recall bias. However, we did address this issue by including the period of the most recent (previous) year in our questionnaire. Although we have endeavored to explain the logical viewpoint behind our findings, a more detailed explanation should be obtained from a qualitative study. Despite the sample size being able to represent secondary school students in Chachoengsao Province, the population of Chachoengsao province may not represent the whole Thai population. Therefore, a nationwide study should be conducted to confirm the problem of internet gaming disorder in Thailand.

## Data Availability

The data sets used during the current study are available from the corresponding author on reasonable request.

## Abbreviations

IGD: the internet gaming disorder
OR: odds ratio
APA: The American Psychological Association
DSM-5: The Diagnostic and Statistical Manual of Mental Disorders, Fifth Edition
DASS-21: The Depression Anxiety Stress Scales
IGD-20 Test: internet gaming disorder test
GPA: grade point average
THB: Thai baht
USD: US dollar
95% CI: 95% confidence interval
